# Artificial Selection of *Gn1a* Plays an Important role in Improving Rice Yields Across Different Ecological Regions

**DOI:** 10.1186/s12284-015-0071-4

**Published:** 2015-12-16

**Authors:** Jie Wang, Huaxue Xu, Nengwu Li, Fengfeng Fan, Liuting Wang, Yingguo Zhu, Shaoqing Li

**Affiliations:** State Key Laboratory of Hybrid Rice; Key Laboratory for Research and Utilization of Heterosis in Indica Rice of Ministry of Agriculture; Engineering Research Center for Plant Biotechnology and Germplasm Utilization of Ministry of Education; College of Life Science, Wuhan University, Wuhan, 430072 China

**Keywords:** Rice, Artificial selection, *Gn1a*, Allele, Association analysis

## Abstract

**Background:**

Rice is one of the most important crops, and it is essential to improve rice productivity to satisfy the future global food supply demands. *Gn1a* (*OsCKX2*), which encodes cytokinin oxidase/dehydrogenase, plays an important role in regulating rice grain yield.

**Results:**

In this study, we analyzed the genetic variation of *Gn1a*, which influences grain yield through controlling the number of spikelets in rice. The allelic variations in the promoter, 5’ untranslated region (UTR) and coding sequence (CDS) of *Gn1a* were investigated in 175 cultivars and 21 wild rice accessions. We found that *Gn1a* showed less sequence variation in the cultivars, but exhibited significant nucleotide diversity in wild rice. A total of 14 alleles, named AP1 to AP14, were identified in the cultivars based on the amino acid divergence of GN1A. Association analysis revealed that the number of spikelets and grain yield were significantly different between the different alleles. Phylogenetic analysis indicated that the three main alleles, AP3, AP8 and AP9, in the cultivars might originate from a common ancestor allele, AP1, in wild rice.

**Conclusions:**

Of these alleles in the cultivars, AP9 was suggested as the best allele in *indica*, as it has shown strong artificial selection in breeding high-yield rice in the past. It might be valuable to explore the high-yield-related alleles of *Gn1a* to develop high-yield rice cultivars in future breeding programs.

**Electronic supplementary material:**

The online version of this article (doi:10.1186/s12284-015-0071-4) contains supplementary material, which is available to authorized users.

## Background

With the rapid decrease in the available farmland and increase in the global population, it is urgent to obtain enough grain production to meet the food supply demands (Weng et al. 2008). With more than 10,000 varieties, rice is a safe and staple food for half of the world’s population (Miura et al. 2010), and thus improving rice productivity is a significant challenge due to the expansion of the world’s population. Rice grain yield is controlled by four basic components, including number of panicles per plant, number of spikelets per panicle, the seed setting rate, and the grain weight, all of which are typical quantitative traits (Xing and Zhang 2010). In the last few decades, a large number of QTLs related to rice grain yield, such as *Gn1a* (Ashikari et al. 2005), *OsSPL14* (Jiao et al. 2010; Miura et al. 2010), *APO1* (Ikeda et al. 2007), *DEP1* (Huang et al. 2009), *OsARG* (Ma et al. 2012), *TAWAWA1* (Yoshida et al. 2013), *OsEBS* (Dong et al. 2013), *DST* (Li et al. 2013) and *PAY1* (Zhao et al. 2015), had been functionally characterized by positional cloning. These important functional genes are usually generated from mutations that occurred in natural populations, such as wild rice and landraces, the ancestors of modern cultivars. Of these, *Gn1a* (*OsCKX2*) is the first gene to be isolated that controls the rice grain yield by regulating cytokinin and the number of spikelets. Reduced expression of *Gn1a* causes cytokinin accumulation in the inflorescence meristems and, consequently, increases the number of spikelets (Ashikari et al. 2005). *Gn1a* plays an important role in regulating rice grain yield. However, the allelic diversity and evolutionary relationship of *Gn1a* in rice populations and the genetic effects of these alleles on grain yield are still unclear. Thus, it is important to explore the potentially favorable alleles of *Gn1a* to improve rice yield when designing strategies in molecular breeding programs.

In this study, the variation and evolutionary pattern of GN1A in wild rice and landraces were investigated. The relationships between the alleles and rice yield components, including the number of spikelets, thousand-grain weight, seed setting rate and effective panicles per plant, were analyzed by association analysis. We found that the high-yield-related alleles of GN1A in the rice populations showed strong artificial selection during rice domestication, which indicated that *Gn1a* played an important role in rice improvements.

## Results

### Nucleotide Variations of *Gn1a*

**Fig. 1:**
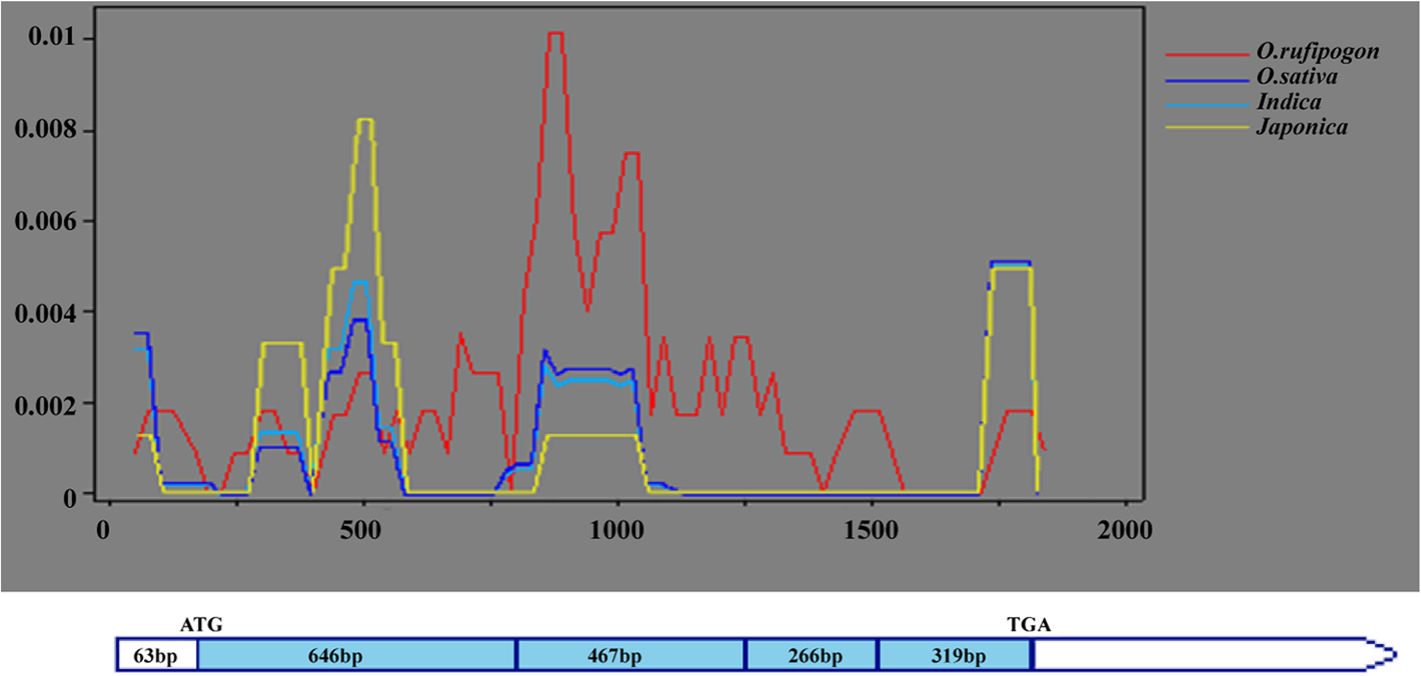
Sliding-window analysis for the *Gn1a* alleles in *O. rufipogon*, *O. sativa*, *indica* and *japonica* cultivars. The genomic structure from the 5’UTR to the stop codon (excluding introns) is shown at the bottom; the blue boxes and white boxes indicate the exons and UTRs. The red, blue, light green and yellow curves represent the *O. rufipogon*, *O. sativa*, *indica* cultivars and *japonica* cultivars, respectively

The *Gn1a* sequences were cloned and sequenced from the cultivar lines and wild rice accessions, which represented a diverse range of spikelet numbers. The 5’UTR and coding frame of 1761 bp were investigated in 175 cultivar lines (Additional file 1: Table S1) and 21 wild rice accessions of *O. rufipogon* (Additional file 2: Table S2). The average number of nucleotide differences per site between any two DNA sequences from the sample population (π) was used to estimate the polymorphisms between *O. sativa* and *O. rufipogon* (Table 1). The nucleotide diversity of *Gn1a* from the *indica* and *japonica* cultivars and *O. rufipogon* were 0.00108, 0.00108 and 0.00203, respectively. The π value of *O. rufipogon* (0.00203) was higher than that of *O. sativa* (0.00110). The sliding-window diagram showed that the majority of the variable sites between *O. sativa* and *O. rufipogon* were located in exon 2 and exon 3 of *Gn1a* (Fig. 1). This finding indicated that the *Gn1a* alleles were more diverse in the wild rice accessions than in the cultivars.

**Table 1:** Polymorphisms and neutrality tests of *Gn1a* in different species

Sample population	π	θw	Tajima's D	Fu and Li's D	Fu and Li's F
*Oryza sativa* (n=175)	0.0011	0.00162	−0.85199	−3.34175*	−2.86108*
*Oryza rufipogon* (n=21)	0.00203	0.00382	−1.79223	−1.27594	−1.66681
*Indica*-like accessions (n=145)	0.00108	0.00168	−0.96016	−3.16946*	−2.79096*
*Japonica*-like accessions (n=32)	0.00108	0.00095	0.41135	1.26945	1.17766

The number in bracket represents the number of corresponding materials. “*” indicates a significant difference at P < 0.05

Based on the nucleotide variations, a total of 17 single nucleotide polymorphisms (SNPs) and five insertion/deletions (indels) of *Gn1a* were detected in *O. sativa*. These SNPs and indels represented 22 combinations of DNA sequence variations and were named A1 to A22 (allele 1 to allele 22, Table 2). Of these, two indels located at site 664 and 667 caused frameshifts and produced premature GN1A proteins; the other two indels in the CDS caused amino acid deletions. Additionally, two synonymous SNPs occurred at site 741 and 828 in the second exon. Previous research showed that there were three SNPs and three indels in the 5’UTR and coding sequence of *Gn1a*, including an 11-bp deletion in the third exon, which generated a large panicle in 5150 (Ashikari et al. 2005). The 11-bp deletion in the third exon was not detected in our tested cultivars, but was detected in the wild rice accession, indicating that the 11-bp deletion in the third exon might originate from *O. rufipogon*. Then, we analyzed the divergence of protein sequence of each allele and found 14 variations in the cultivars at protein level, which were renamed as alleles AP1 to AP14 (alleles for protein 1 to protein 14, Table 3). Among these alleles, AP5, AP10 and AP13 formed premature proteins, and the others only changed the number of amino acids.

**Table 2:** Nucleotide variations in the 5’UTR and CDS of *Gn1a*

Alleles	−124	−31	−7	161	236	319	353	661	663	664	665	666	667	688	741	781	782	828	885	910	984	1611
A1	A	A	16bp	G	6bp	G	A	G	G	A	T	G	G	G	C	G	C	C	G	G	3bp	T
A2	A	A	16bp	C	-	G	G	G	G	A	T	G	G	G	C	G	C	C	G	G	3bp	T
A3	A	A	16bp	C	12bp	G	G	G	G	A	T	G	G	G	C	G	C	C	G	G	3bp	T
A4	A	A	16bp	C	12bp	T	G	G	G	A	T	G	G	G	C	G	C	C	G	G	3bp	T
A5	A	A	16bp	C	12bp	T	G	G	G	A	T	G	G	G	C	G	C	C	G	G	3bp	G
A6	A	A	16bp	C	6bp	G	A	G	G	A	T	G	G	G	C	G	C	C	G	G	3bp	T
A7	A	A	-	C	12bp	G	G	G	G	A	T	G	G	G	C	G	C	C	G	G	3bp	T
A8	G	A	-	C	-	G	G	G	G	A	T	G	G	G	C	G	C	C	G	G	3bp	T
A9	A	A	-	C	-	G	G	G	G	A	T	G	G	G	A	G	C	A	G	G	3bp	G
A10	A	A	-	C	-	G	G	G	G	A	T	G	G	G	C	G	C	C	G	G	3bp	G
A11	A	A	-	G	6bp	G	A	G	G	A	T	G	G	G	C	G	C	C	G	G	3bp	T
A12	A	A	-	C	12bp	G	G	G	G	A	T	G	G	G	C	G	C	C	G	G	3bp	G
A13	A	A	-	C	6bp	G	G	G	G	A	T	G	G	G	C	G	C	C	G	G	3bp	T
A14	G	A	-	G	6bp	G	A	G	G	A	T	G	G	G	C	G	C	C	G	G	3bp	T
A15	G	A	-	C	12bp	T	G	G	G	A	T	G	G	G	C	G	C	C	G	G	3bp	T
A16	A	A	-	C	-	G	G	G	G	-	T	G	G	G	C	G	C	C	G	G	3bp	G
A17	A	A	16bp	C	12bp	T	G	G	G	-	T	G	G	G	C	G	C	C	G	G	3bp	G
A18	A	A	16bp	C	12bp	T	G	G	G	A	T	G	G	A	C	G	C	C	G	G	3bp	G
A19	A	A	-	C	-	G	G	G	G	-	T	G	G	G	A	G	C	A	G	G	3bp	G
A20	A	A	-	C	-	G	G	T	C	A	C	C	-	G	A	T	T	A	C	T	3bp	G
A21	A	A	-	C	-	G	G	G	G	A	T	G	G	G	A	G	C	A	G	G	-	G
A22	A	C	-	C	-	G	G	G	G	A	T	G	G	G	C	G	C	C	G	G	3bp	G

The number in the first line indicates the variable sites in the 5’UTR and CDS (excluding the introns). “-” indicated the deletions at the corresponding sites

**Table 3:** Amino acid variations in the different alleles in *O. sativa*

Haplotype	Frequency	54	79	107	118	221	230	328	537
AP1 (A12)	1.1%	A	AAAA	A	R	EMV	A	S	K
AP2 (A3, A7)	8.0%	A	AAAA	A	R	EMV	A	S	N
AP3 (A5)	14.9%	A	AAAA	S	R	EMV	A	S	K
AP4 (A4, A15)	3.4%	A	AAAA	S	R	EMV	A	S	N
AP5 (A17)	1.1%	A	AAAA	S	R	EM&			
AP6 (A13)	0.6%	A	AA--	A	R	EMV	A	S	N
AP7 (A6)	0.6%	A	AA--	A	H	EMV	A	S	N
AP8 (A9, A10, A22)	32.0%	A	----	A	R	EMV	A	S	K
AP9 (A2, A8)	27.4%	A	----	A	R	EMV	A	S	N
AP10 (A16, A19)	1.7%	A	----	A	R	EM&			
AP11 (A1, A11, A14)	6.9%	G	AA--	A	H	EMV	A	S	N
AP12 (A21)	1.1%	A	----	A	R	EMV	A	-	K
AP13 (A20)	0.6%	A	----	A	R	YT&			
AP14 (A18)	0.6%	A	AAAA	S	R	EMV	T	S	K

The number in the first line indicated the variable amino acid sites of the GN1A protein; the *Gn1a* alleles are in brackets

### Phenotypic Analysis of the GN1A Alleles

**Fig. 2:**
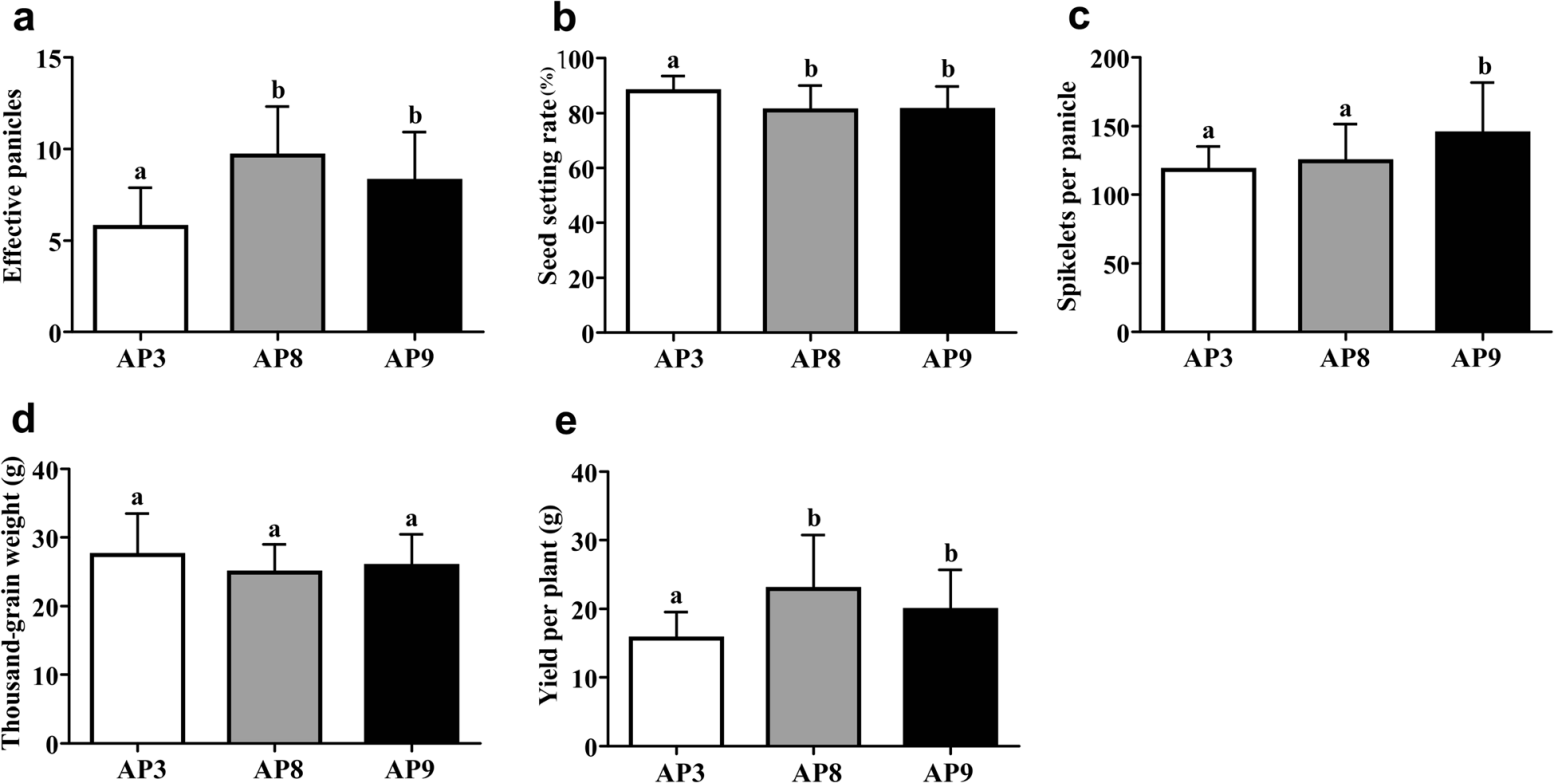
Comparisons of the plant yield and agronomic traits of AP3, AP8 and AP9 in *indica* varieties. **a** Comparison of the effective panicles per plant between AP3, AP8 and AP9. **b** Comparison of the seed setting rate among AP3, AP8 and AP9. **c** Comparison of the spikelets per panicle among AP3, AP8 and AP9. **d** Comparison of the thousand-grain weight among AP3, AP8 and AP9. **e** Comparison of the yield per plant among AP3, AP8 and AP9. All data are presented as the means ± SD. The letters above the bars are ranked by Duncan test at *P* < 0.05; the same letters indicate no significant differences, and different letters indicate significant differences

As *Gn1a* functions to control rice yield by regulating the number of spikelets, we analyzed the relationship between the AP alleles and yield components, including the effective panicles per plant, thousand-grain weight, seed setting rate, and spikelets per panicle. Because alleles AP3, AP8 and AP9 represented the highest frequency, over 10 % in the *indica* subpopulation (Table 3), they were used for the association analysis. The result showed that there was a significant difference in the effective panicles per plant between AP3 and the other two alleles, but there was no difference between AP8 and AP9 (Fig. 2a). There was a significant difference in the seed setting rate between AP3 and both AP8 and AP9, although no significant difference was detected between the AP8 and AP9 alleles (Fig. 2b). For the spikelets per panicle, the means of AP3, AP8 and AP9 were 119.50, 124 and 144.73, respectively, which showed an extremely significant difference (*P* < 0.01) between AP9 and AP3, and a significant difference (*P* < 0.05) between AP8 and AP9 (Fig. 2c). These results suggested that cultivars with the AP9 allele had a higher number of spikelets per panicle compared to AP3 and AP8, without changing the thousand-grain weight (Fig. 2d). However, there was no significant difference in the grain yield per plant between AP8 and AP9 (Fig. 2e) because AP8 had a relatively higher number of panicles per plant than AP9. From the results above, we concluded that AP9 was more prevalent than AP8 in cultivars because it produced more spikelets per panicle.

### Expressional Analysis of the *Gn1a* Alleles

**Fig. 3:**
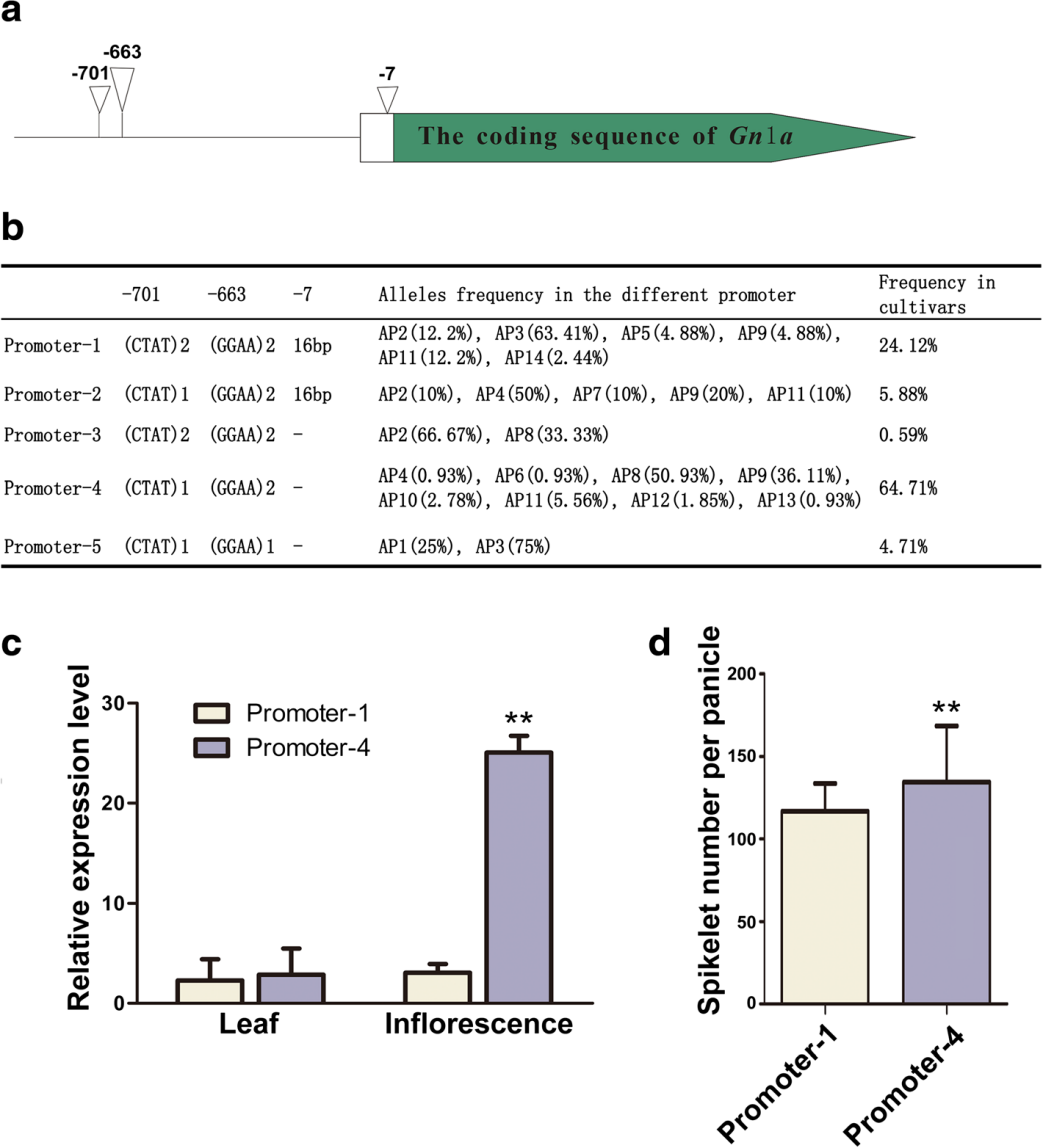
The structure and transcription level of the different promoters. **a** The variation sites where the different deletions occur; “∇” indicates the deletions. **b** Different types of promoters; “-” indicates a 16-bp deletion. The percentage in brackets located after the alleles indicates the frequency of the alleles in the corresponding promoters. **c** Relative expression level between Promoter-1 and Promoter-4 in young leave and inflorescences. “**” indicates a significance difference (*P* < 0.01). **d** Comparison of the number of spikelets per panicle between Promoter-1 and Promoter-4. “**” indicates a significance difference (*P* < 0.01)

To determine whether the *Gn1a* transcript was closely associated with the number of spikelets, we analyzed the promoter and 5’UTR of *Gn1a,* which contains 1500 bp upstream ATG of *Gn1a* in the cultivars, and found two indels at −663 bp and −701 bp, as well as a 16-bp deletion in the 5’UTR (Fig. 3a). Based on these three indels, five combinations named Promoter-1 to Promoter-5 were detected, and the frequency of Promoter-1 (24.12 %) and Promoter-4 (64.17 %) was apparently higher than the other alleles (Fig. 3b). Promoter-1 contained 6 alleles, of which allele AP3 was the major allele, accounting for 63.41 %. Promoter-4 was comprised of 8 alleles, among which AP8 and AP9 were the most frequent alleles and accounted for 50.93 % and 36.11 %. The other alleles were less than 6 %.

AP3, AP8 and AP9 were the popular alleles in rice populations, which indicated that Promoter-1 and Promoter-4 were the two major types of promoters in the cultivars. Then, we assayed the expression level of *Gn1a* between Promoter-1 and Promoter-4 in young leaves and inflorescences in the cultivars. The qRT-PCR analysis showed that there was no significant difference in either promoter in the young leaves, but the expression level of Promoter-4 was 20-fold higher than that of Promoter-1 in the inflorescences (Fig. 3c). Furthermore, the number of spikelets per panicle between Promoter-1 and Promoter-4 were analyzed, and the results indicated that Promoter-4 generated more spikelets than Promoter-1 (Fig. 3d). These results suggested that the protein sequence and expression level of *Gn1a* might affect the number of grains, as cultivars harboring AP9 could produce more grains than cultivars with AP8.

### Distribution and Evolutionary Pattern of the GN1A Alleles in the *Oryza* Genus

To understand the general status of the GN1A alleles in the *Oryza* genus, we further investigated the distribution of the GN1A alleles in the wild rice *O. rufipogon*, and found that, with the exception of AP1, AP3 and AP8, nine new alleles, named AP15 to AP23, were detected in *O. rufipogon* (Additional file 3: Table S3). Of these, 7 out of 21 wild rice accessions harbored AP1, which indicated that AP1 might be the original allele in the *Oryza* genus*.*

**Fig. 4:**
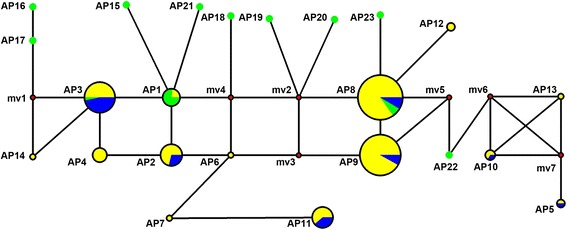
The phylogenetic network for the 23 alleles of GN1A between wild and cultivated rice species. Each circle represents an allele. The size of the circles indicates the frequency of each allele. The green, yellow and blue circles indicate the allelic distribution in the wild rice, *indica* and *japonica* species, respectively. The red median vectors (mv) represent a hypothesized sequence that is required to connect the existing sequences within the network with maximum parsimony. The solid lines represent one mutation step that interconnects adjacent alleles

A phylogenetic network for alleles of GN1A was constructed based on the amino acid sequence of the 23 alleles in *O. sativa* and *O. rufipogon* (Fig. 4). Using this network, we found that AP3, AP8 and AP9 were the most popular alleles in the cultivars. Interestingly, AP8 and AP9 were primarily distributed in *indica*, and rarely found in *japonica* rice. However, the ratios of AP3, AP5, AP10 and AP15 were not apparently different between *indica* and *japonica*, which reflected a strong artificial selection during rice domestication. Actually, there was only a single amino acid variation at position 537 between AP8 and AP9 (Table 3), implying their tight evolutionary relationship.

**Fig. 5:**
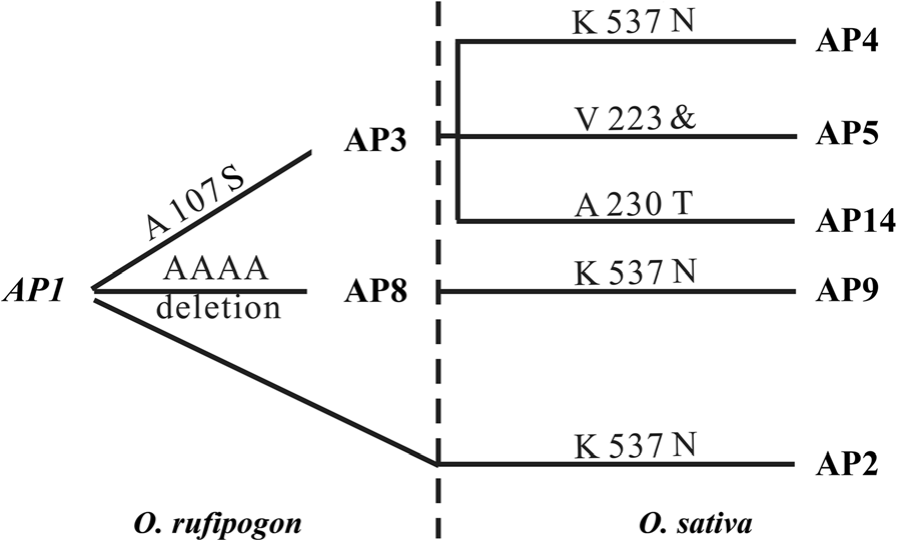
Evolutionary pattern of the most frequent alleles of GN1A in the *Oryza* genus. Substitution and deletion events occurred above each line, and the substitution of K 537 to N played an important role in the evolutionary process from wild rice to the common cultivars

Based on the frequency and distribution of the alleles in the cultivars and *O. rufipogon*, we assumed that AP1 might be the original allele, as it had the highest frequency in wild rice. Thus, we proposed a schematic diagram to describe the possible evolutionary pattern of the main alleles of *Gn1a* (Fig. 5). The model suggested that in the *O. rufipogon* population, the primitive allele AP1 might have evolved to AP3 and AP8, respectively, by the loss of two or three alanine repeats at the amino acid 79, as the majority of wild rice encoded the amino acid 79 by four-alanine repeats. Then, the lysine 537 of AP1 and AP8 had mutated to asparagine in AP2 and AP9, respectively. AP9 was then strongly selected during domestication and ultimately became the most prevalent allele indifferent rice populations because of its larger panicle.

### The Artificial Selection Pattern of *Gn1a*

The Tajim’s D (Tajima 1989), Fu and Li’s D and F tests (Fu 1997) are usually employed to assess the trends of artificial selection of genes in plant populations. To understand whether the evolutionary process of *Gn1a* was affected by artificial selection, we investigated the selection pattern using the Tajima’s D, Fu and Li’s D and F tests in the different rice species (Table 1). The values of Fu and Li’s D and F tests were −3.34715 and −2.86108 in *O. sativa*, which were significantly negative values compared to *O. rufipogon*. This result indicated that the excess low-frequency polymorphisms of the alleles in *O. sativa* deviated from the neutral expectation, implying there was a strong artificial selection of *Gn1a* in cultivar populations (Du et al. 2011).

Then we investigated the alleles of GN1A in the pedigree of Guichao 2, a high grain-yield cultivar bred in China in the 1980s-1990s, as a model to test how artificial selection influences the distribution of the GN1A alleles in breeding programs. In this pedigree, all of the tested accessions carried allele AP9, with the exception of Qingnong’ai (Additional file 4: Figure S1). This result implied that when another allele was introduced in the pedigree, the excellent AP9 allele was still selected in the offspring, namely artificial selection accelerated the concentration of the large panicle AP9 allele of GN1A in cultivar populations. This was consistent with the Tajim’s D, Fu and Li’s D and F tests, and provided us a new insight into the evolutionary pattern of GN1A during rice domestication. These high-yield-related alleles could facilitate the development of high-yield rice in future breeding programs.

### Geographic Distribution of the GN1A Alleles

**Fig. 6:**
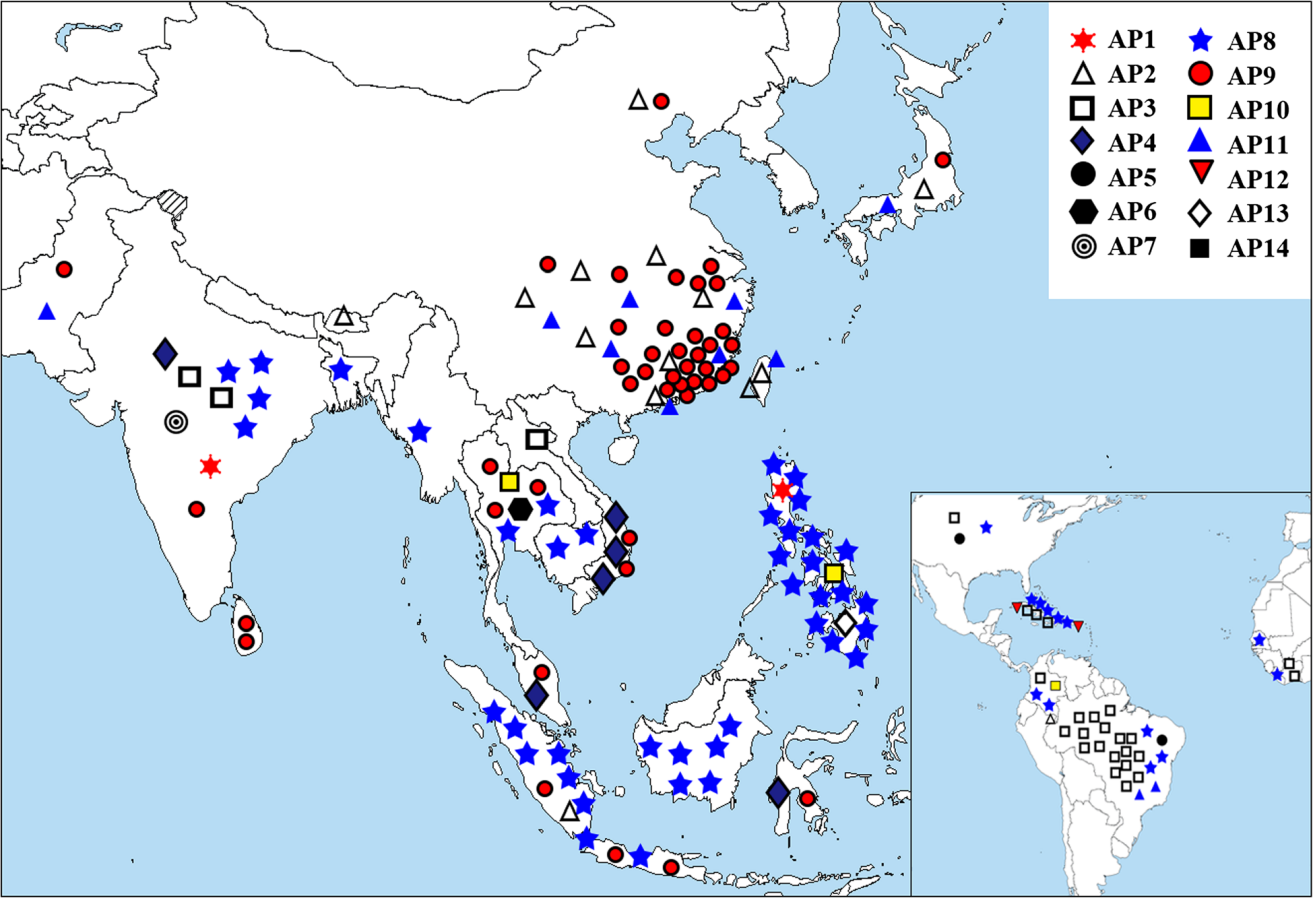
Geographic distribution of the 14 different alleles of GN1A. Symbols with different colors indicate different alleles, and each symbol represents one accession

We analyzed the regional distribution of the GN1A alleles in the cultivars to explore the relationship between the evolutionary pattern and geographic distribution of the GN1A alleles (Fig. 6). Interestingly, these alleles showed a strong distinctive geographic character; AP3, AP8 and AP9 spread over seven countries in different continents. AP3 was primarily located in Brazil, AP8 was primarily located in Indonesia and the Philippines, and AP9 was primarily concentrated in China. The other alleles were mostly limited to a single area; for example AP6, AP7, AP13 and AP14 were found only in Thailand, India, the Philippines and Colombia, respectively. These results indicated that AP3 had primarily concentrated in the Americas, and AP8 and AP9 were the most frequent alleles in Asia.

## Discussion

Plant hormones, including auxin (Wang et al. 2014; Zhang et al. 2015a; Zhao et al. 2015), gibberellins (Asano et al. 2011; Wu et al. 2014; Zhang et al. 2015b), abscisic acid (Santiago et al. 2009; Zhang et al. 2015b), ethylene (Strader et al. 2010; Yin et al. 2015), cytokinin (Ashikari et al. 2005; Bartrina et al. 2011; Li et al. 2013) and brassinosteroids (Kim et al. 2012; Sun et al. 2015), are important for plant growth and development. Cytokinin has been considered to be an important factor for modulating inflorescence meristem development (Nishimura et al. 2004; Riefler et al. 2006), and, hence, plays a crucial role in determining the rice grain yield. *Gn1a*, which encodes cytokinin oxidase/dehydrogenase, plays a critical role in regulating the number of grains per panicle and rice yield. It has been verified that pyramid *Gn1a* and *sd1* could increase the grain number by 23 %, without changing the grain size (Ashikari et al. 2005). It will be important to explore the high-yield-related alleles of *Gn1a* in breeding programs as effective strategy to create high-yield rice to satisfy the daily requirements of the increasing global population.

In this study, we analyzed the sequences of GN1A alleles in cultivars and common wild rice accessions, and found that AP3, AP8 and AP9 were the most frequent alleles in the cultivars. Of these, AP9 was superior to AP3 and AP8 with regard to the number of spikelets. Compared to AP3, both AP8 and AP9 had a four-alanine deletion in the first exon of GN1A. However, there was no significant difference in the number of spikelets between AP3 and AP8, which indicated that these four tandem alanine repeats in the first exon did not have a special function in spikelet development. When lysine 537 in AP8 was substituted to asparagine in AP9, the spikelets per panicle increased. Thus, we presumed that the substitution of amino acid 537 in GN1A exerted a critical function on determining the grains number of rice panicles. Although AP9 promoted an increased number of spikelets, the grain yield for the AP9 allele did not increase significantly compared to AP8. It was possibly that other genes related to effective panicles and grain weight might compromise the effects of AP9 in a complicated genetic background. To accurately understand the effects and the molecular mechanism of AP8 and AP9 on rice productivity, a set of nearly isogenic lines containing each of these GN1A alleles should be constructed to exclude the background noise in rice breeding programs.

Compared to the other alleles, AP1 was the most frequently detected allele in the common wild rice accessions in this study, and the frequency reached 33.3 % in *O. rufipogon*. This result implied that AP1 was likely the original allele in *O. rufipogon*. Based on the protein sequence, we deduced that both AP3 and AP8 might originate from AP1 because there was only one variant site between AP1 and AP3/AP8. Interestingly, there was only one amino acid difference between AP8 and AP9, which substituted lysine 537 in AP8 and for asparagines 537 in AP9. Because asparagines 537 was not detected in *O. rufipogon*, we proposed that AP9 was directly mutated from AP8 in the cultivars. AP3 might further differentiate into other alleles, such as AP4, AP5 and AP14. However, it was still difficult for us to deduce the original ancestor of the other rare alleles in the rice accessions because of the limited wild rice accession samples investigated in this study. More wild rice accessions in the *Oryza* genus need to be analyzed to elucidate the detailed evolutionary pattern of the GN1A alleles.

## Conclusions

In this study, we characterized allele AP9 as the most frequent allele, due to the large panicle and high yield per plant, which may facilitate the development of high-yield rice in future breeding programs.

## Methods

### Plant Materials and Trait Analysis

The 175 cultivar lines and 21 wild rice accessions of *O. rufipogon*, from 19 countries representing a diverse range of grains number and plant yield, were collected from the International Rice Research Institute (IRRI) and our laboratory were grown under normal field conditions on the island of Hainan, China and the E’Zhou experiment station in E’Zhou (Hubei Province, China) from 2012 to 2014. Most cultivars are landraces from rice-growing regions in China, Indonesia and the Philippines. Ten plants with a density of 16.5 cm × 26.5 cm were planted in a row and 5 rows of each accession were planted.

The number of effective panicles per plant was counted as the number of tillers bearing more than 5 grains. The number of spikelets per panicle was measured as the total number of spikelets divided by the effect panicles. The yield per plant was scored as the total weight of the grains per plant. The thousand-grain weight was scored as the yield per plant divided by the total number of grains multiplied by 1000. The seed setting rate was tested as the number of grains per panicle divided by the the number of spikelets per panicle. All traits were measured from 5 randomly sampled plants for each accession after harvest.

### DNA Extraction, PCR Amplification and Sequencing

The genomic DNA was extracted from fresh leaves using cetyltrimethyl ammonium bromide (Murray and Thompson 1980). The primers that were used to amplify the promoter, 5’UTR and CDS were designed using the *Gn1a* allele of Nipponbare. The PCRs were performed under the standard PCR protocols, and the 20 μl PCR system contained 50 ng of the genomic DNA template, 0.5 μl of the forward and reverse primers (both 10 μM), 1.6 μl of 2.5 mM dNTPs, 1.6 μl of 25 mM MgCl_2_, 0.3 μl of 5 U/μlrTap polymerase and 2 μl 10 × rTap buffer. The 20 μl PCR products were recycled using a TIANGEN recycling kit, and then ligated into the pGEM18-T Easy Vector. Each independent plasmid DNA was sequenced by Tsingke (Wuhan, China). The correct DNA sequence was selected after comparing three plasmids from each accession. All primers used for PCR amplification were presented in Additional file 5: Table S4.

### DNA Sequences Analysis and Alleles Analysis

The DNA sequences from the different *Oryza* accessions were aligned using CLUSTAL W version 2.0 and adjusted manually with BIOEDIT. Two measures of nucleotide variability, the average number of nucleotide differences per site (π) (Beverley et al. 1987) and nucleotide diversity based on the proportion of segregating sites (θω) (Watterson 1975), were obtained using DNASP v5.0 (Librado and Rozas 2009). The selection tests, including Tajima’s D test (Tajima 1989), Fu and Li’s D and F tests, were performed using DNASP v5.0. The sliding-window method was employed to value the polymorphisms across the coding sequence of *Gn1a*, using a window size of 100 and a step size of 20, in which pairwise insertions and deletions were removed via DNASP v5.0. The allelic diversity analysis was performed using BIOEDIT and the MAGE 5.0 program (Tamura et al. 2011). The phylogenetic network was constructed by Network 4.611 according to the corresponding user guide.

### RNA Isolation and RT-PCR

The total RNA was prepared from the fresh leaves at the seedling stage and inflorescences at the young panicle stage using an RNAprep pure Plant Kit (Invitrogen, Carlsbad, CA, USA). The first-strand cDNA was synthesized from the total RNA using a Superscript II RT Kit (Invitrogen, Carlsbad, CA, USA). All RT-PCRs were performed using the first-strand cDNA as the template; 35 PCR cycles were used to amplify the *Gn1a* transcripts, and 25 PCR cycles were used to amplify the *Actin* transcripts. Real-time quantitative PCR was performed in the Roche Light Cycler 480 PCR system according to the manufacturer’s instruction. The rice *Actin* gene was used as the internal control, and three technical replicates were performed for each sample. The expression level was calculated using the relative quantification method.

### Statistical Analysis

The statistical analyses were performed using the SPSS software, and the comparisons of the number of spikelets per panicle, thousand-grain weight, seed setting rate, effective panicles and plant yield among the alleles were calculated by one-way ANOVA. Nonparametric tests were performed if there was no homogeneity of variance. If the results of the analysis were significant (*P* < 0.05), Duncan’s multiple range test was used as a post hoc test for multiple comparisons. The histograms were constructed using the GraphPad Prism 5 software and modified by Adobe Photoshop CS5. All tests were performed at least three times.

## Additional files

Additional file 1: Table S1. Germplasm variety information and their alleles of *Gn1a.* “NO” in the first line indicates the serial number in the field; SPP, TGW, PY, SSR and EP indicate the number of spikelets per panicle, thousand-grain weight, seed setting rate and effective panicles per plant, respectively; “/” indicates that the data were not available. (DOC 266 KB) Additional file 2: Table S2. Basic information on the wild rice varieties. The number ahead of each material indicated the serial number in the field in 2012, and the alleles were established based on the different amino acid sequences among the wild rice varieties. (DOC 38 KB) Additional file 3: Table S3. Amino acid variations of the different alleles in *O. rufipogon.* The number in the first line indicates the variable amino acid sites of the GN1A protein and the GN1A alleles are in brackets; “-” indicates the deletion of the corresponding amino acid, and “&” indicates the stop codon of the protein. (DOC 68 kb) Additional file 4: Figure S1. Allele analysis of the pedigree of Guichao 2. The colored materials were selected for this study; the green varieties harbored the AP9 allele and the yellow varieties contained the AP2 allele. (DOC 157 kb) Additional file 5: Table S4. Primers used in this study. (DOC 30 kb)
