# Supplementary material for: Artificial Selection of Gn1a Plays an Important role in Improving Rice Yields Across Different Ecological Regions
Source: Rice (N Y). 2015 Dec 16;8:37. doi: 10.1186/s12284-015-0071-4 (PMC4681714; doi:10.1186/s12284-015-0071-4)
Supplement: Additional file 1: Table S1. — Germplasm variety information and their alleles of Gn1a. “NO” in the first line indicates the serial number in the field; SPP, TGW, PY, SSR and EP indicate the number of spikelets per panicle, thousand-grain weight, seed setting rate and effective panicles per plant, respectively; “/” indicates that the data were not available. (DOC 266 KB) [file 12284_2015_71_MOESM1_ESM.doc]

**Additional file 1: Table S1.**

| **NO** | **Variety** | **Alleles of DNA** | **Alleles of protein** | **SPP** | **PY(g)** | **TGW(g)** | **RRS** | **EP** | **suppopulation** | **Resource** |
| --- | --- | --- | --- | --- | --- | --- | --- | --- | --- | --- |
| 1 | Pratao tipo guedes | A5 | AP3 | 138 | 22.66 | 24.8 | 86.26% | 8 | *indica* | Brazil |
| 2 | Linea 9 | A9 | AP8 | 121 | 37.31 | 22.9 | 82.44% | 16 | *indica* | Cuba |
| 3 | Sitio boa vista | A5 | AP3 | 84 | 17.10 | 31.8 | 90.87% | 7 | *japonica* | Brazil |
| 4 | Bkn6625-109-1 | A9 | AP8 | 123 | 45.16 | 31.3 | 88.37% | 13 | *indica* | Thailand |
| 5 | Itarare | A10 | AP8 | 121 | 18.09 | 23.7 | 84.78% | 8 | *japonica* | Brazil |
| 6 | IR 9678-3-8-8-1 | A10 | AP8 | 133 | 15.65 | 22.3 | 87.83% | 6 | *indica* | Philippines |
| 7 | IR 442-2-58 | A9 | AP8 | 107 | 21.30 | 23.1 | 66.66% | 13 | *indica* | Philippines |
| 9 | Iac703-73 | A5 | AP3 | 106 | 10.83 | 34.8 | 86.31% | 3 | *indica* | Brazil |
| 10 | Hmong dam | A5 | AP3 | 89 | 11.99 | 35.3 | 95.06% | 4 | *indica* | Laos |
| 11 | Lambari | A5 | AP3 | 98 | 18.99 | 30.4 | 90.48% | 7 | *japonica* | Brazil |
| 12 | Tos7582 | A5 | AP3 | 105 | 12.77 | 24.9 | 86.15% | 6 | *indica* | Ivory coast |
| 13 | Daharnagra | A9 | AP8 | 112 | 19.93 | 20.7 | 83.26% | 10 | *indica* | India |
| 14 | Tondano | A10 | AP8 | 124 | 22.69 | 26.6 | 92.72% | 7 | *indica* | Indonesia |
| 15 | Singgal(c-5862) | A10 | AP8 | 167 | 27.27 | 28.3 | 72.31% | 8 | *indica* | Indonesia |
| 16 | Cateto(moti) | A5 | AP3 | 80 | 14.81 | 26.9 | 93.64% | 7 | *japonica* | Brazil |
| 17 | Gulfmont | A5 | AP3 | 125 | 20.64 | 25.5 | 86.09% | 8 | *indica* | United States |
| 18 | S.ir880-c9 | A21 | AP12 | 120 | 26.96 | 26.5 | 88.11% | 12 | *indica* | Cuba |
| 19 | Japones gigante sel.299 | A1 | AP11 | 101 | 22.39 | 27.4 | 78.20% | 10 | *japonica* | Brazil |
| 20 | Mexico 221 | A10 | AP8 | 123 | 16.77 | 24.7 | 79.00% | 7 | *indica* | Cuba |
| 21 | Amarelinho | A5 | AP3 | 125 | 18.46 | 30.8 | 92.25% | 4 | *indica* | Brazil |
| 22 | Jc43 | A10 | AP8 | 110 | 20.19 | 22.8 | 83.06% | 10 | *indica* | India |
| 23 | IR 262-43-8-11 | A22 | AP8 | 118 | 21.77 | 25.4 | 68.47% | 11 | *indica* | Philippines |
| 24 | Colombia 3 | A19 | AP10 | 167 | 16.56 | 19.5 | 72.38% | 7 | *indica* | Colombia |
| 25 | Sequeiro ou chapa deiro | A17 | AP5 | 96 | 15.56 | 31.5 | 91.14% | 6 | *japonica* | Brazil |
| 26 | Prae 104-4-78 | A16 | AP10 | 164 | 25.94 | 37.4 | 79.17% | 5 | *japonica* | Thailand |
| 27 | Douradinho(bold) | A5 | AP3 | 140 | 17.23 | 21.6 | 81.79% | 7 | *indica* | Brazil |
| 28 | B 1050c-mr-7-3 | A9 | AP8 | 157 | 25.54 | 27.3 | 74.32% | 8 | *japonica* | Indonesia |
| 29 | Tos7580 | A5 | AP3 | 123 | 9.11 | 27.1 | 81.88% | 3 | *indica* | Ivory coast |
| 30 | B 3753-7-pn-4-1 | A9 | AP8 | 99 | 21.07 | 23.7 | 76.84% | 12 | *japonica* | Indonesia |
| 31 | IR 1917-3-19-2 | A10 | AP8 | 116 | 21.15 | 19.0 | 87.52% | 11 | *indica* | Philippines |
| 32 | Seleccion 136 | A10 | AP8 | 64 | 10.96 | 24.5 | 90.79% | 8 | *indica* | Cuba |
| 33 | Come-cru btanco | A5 | AP3 | 129 | 20.93 | 28.9 | 92.92% | 5 | *japonica* | Brazil |
| 34 | Come cru blanco | A5 | AP3 | 160 | 14.09 | 27.7 | 93.47% | 3 | *japonica* | Brazil |
| 35 | Ica 10 t 112-7 p 5 t 1 p 2 t | A5 | AP3 | 152 | 15.13 | 20.5 | 80.57% | 5 | *indica* | Colombia |
| 36 | B 2266b-cw-16-2-1 | A9 | AP8 | 143 | 14.07 | 26.4 | 91.60% | 5 | *indica* | Indonesia |
| 37 | Jc130 | A5 | AP3 | 136 | 12.47 | 12.7 | 78.41% | 8 | *japonica* | India |
| 38 | Hai | A10 | AP8 | 73 | 10.19 | 28.5 | 85.52% | 6 | *indica* | Laos |
| 39 | IR 2003-p16-7-1-2 | A10 | AP8 | 114 | 21.28 | 20.7 | 70.94% | 13 | *indica* | Philippines |
| 40 | Conquista | A5 | AP3 | 93 | 8.70 | 29.2 | 87.48% | 4 | *japonica* | Brazil |
| 41 | Silvanoti | A9 | AP8 | 150 | 25.20 | 26.9 | 89.45% | 7 | *indica* | Brazil |
| 42 | Ligeirinho | A5 | AP3 | 129 | 13.24 | 28.6 | 89.67% | 4 | *japonica* | Brazil |
| 43 | Seleccion 123 | A21 | AP12 | 102 | 17.59 | 21.6 | 88.50% | 9 | *indica* | Cuba |
| 44 | Seleccion 132 | A9 | AP8 | 117 | 20.23 | 25.0 | 90.71% | 8 | *indica* | Cuba |
| 45 | IR 4472-53-257-4 | A10 | AP8 | 106 | 23.13 | 31.8 | 71.00% | 10 | *indica* | Philippines |
| 46 | Iet714 | A10 | AP8 | 108 | 20.87 | 31.4 | 71.15% | 10 | *indica* | India |
| 47 | Colombia 2 | A9 | AP8 | 168 | 21.52 | 20.8 | 76.85% | 8 | *indica* | Colombia |
| 49 | IR 9673-8-8-6 | A10 | AP8 | 125 | 28.73 | 24.5 | 74.10% | 13 | *indica* | Philippines |
| 50 | Riz aile | A5 | AP3 | 126 | 18.44 | 30.9 | 94.86% | 5 | *indica* | Brazil |
| 51 | Kn1b-361-8-6-9-4-5 | A9 | AP8 | 150 | 36.05 | 28.9 | 83.06% | 10 | *indica* | Indonesia |
| 52 | Bogo wonto | A10 | AP8 | 97 | 19.28 | 26.3 | 86.85% | 9 | *indica* | Indonesia |
| 53 | 122 | A10 | AP8 | 89 | 16.58 | 27.9 | 71.22% | 9 | *indica* | Thailand |
| 54 | Batatais | A5 | AP3 | 114 | 17.00 | 31.0 | 90.81% | 5 | *indica* | Brazil |
| 55 | Arroz bolito italica | A5 | AP3 | 111 | 19.57 | 28.1 | 93.81% | 7 | *indica* | Cuba |
| 56 | Hg35-O.glaberrima | A10 | AP8 | 158 | 48.25 | 38.3 | 88.46% | 9 | *indica* | Senegal |
| 57 | IR 1561-250-2-2 | A9 | AP8 | 100 | 21.01 | 19.9 | 75.24% | 14 | *indica* | Philippines |
| 58 | 49269 | A10 | AP8 | 102 | 27.00 | 24.7 | 77.78% | 15 | *indica* | Indonesia |
| 59 | B 922c-mr-118 | A9 | AP8 | 107 | 23.17 | 25.6 | 87.31% | 10 | *indica* | Indonesia |
| 60 | IR 883-12-2-1-3 | A10 | AP8 | 187 | 21.29 | 20.2 | 73.38% | 8 | *indica* | Philippines |
| 61 | Douradinho(bold) | A5 | AP3 | 111 | 14.45 | 28.9 | 96.77% | 5 | *indica* | Brazil |
| 62 | Iac 165 | A5 | AP3 | 93 | 12.42 | 34.7 | 85.56% | 5 | *indica* | Brazil |
| 63 | IR 9690-1-1-1-7 | A10 | AP8 | 117 | 28.70 | 26.3 | 77.90% | 12 | *indica* | Philippines |
| 64 | IR 3273-348-1-6 | A10 | AP8 | 113 | 19.44 | 27.6 | 69.13% | 9 | *indica* | Philippines |
| 65 | Seleccion 102 | A9 | AP8 | 95 | 30.68 | 27.5 | 90.28% | 13 | *indica* | Cuba |
| 66 | IR 2071-588-2-5-1 | A10 | AP8 | 130 | 29.53 | 22.2 | 76.37% | 13 | *indica* | Philippines |
| 67 | B 2362-6-2 | A10 | AP8 | 122 | 25.49 | 22.4 | 82.11% | 11 | *indica* | Indonesia |
| 68 | IR 1541-76-3-3 | A9 | AP8 | 130 | 29.48 | 21.8 | 78.15% | 13 | *indica* | Philippines |
| 69 | 49268 | A10 | AP8 | 115 | 10.27 | 20.5 | 43.57% | 10 | *indica* | Indonesia |
| 70 | IR 946-52-2-1-3-3 | A10 | AP8 | 122 | 24.58 | 24.4 | 85.75% | 10 | *indica* | Philippines |
| 71 | B 5711 A 1-18-6 | A17 | AP5 | 137 | 19.22 | 18.7 | 82.83% | 8 | *indica* | United States |
| 72 | Jc117 | A10 | AP8 | 160 | 18.51 | 22.5 | 77.26% | 7 | *indica* | India |
| 73 | Singkarak | A9 | AP8 | 141 | 19.92 | 22.0 | 82.33% | 8 | *indica* | Indonesia |
| 74 | Pusu | A10 | AP8 | 200 | 17.27 | 24.4 | 88.44% | 4 | *japonica* | Indonesia |
| 75 | Colombia 1 | A9 | AP8 | 181 | 23.05 | 17.2 | 88.61% | 8 | *indica* | Colombia |
| 76 | Iac1278 | A10 | AP8 | 129 | 24.28 | 28.6 | 85.78% | 8 | *indica* | Brazil |
| 77 | Ligeiro | A5 | AP3 | 128 | 10.53 | 22.7 | 83.62% | 4 | *japonica* | Brazil |
| 78 | Chinsaba | A10 | AP8 | 165 | 16.33 | 20.1 | 74.08% | 7 | *indica* | Myanmar |
| 79 | Estirpe abreu 14-3 | A1 | AP11 | 129 | 26.19 | 27.0 | 80.48% | 9 | *japonica* | Brazil |
| 80 | IR 2003-p5-3-3 | A10 | AP8 | 122 | 17.24 | 22.9 | 75.85% | 9 | *indica* | Philippines |
| 81 | IR 4472-53-10-8-1-2 | A10 | AP8 | 122 | 22.19 | 29.3 | 86.07% | 8 | *indica* | Philippines |
| 82 | Arc 14914 | A12 | AP1 | 137 | / | / | 94.99% | 9 | *indica* | India |
| 83 | Dhalasaita | A10 | AP8 | 186 | 24.36 | 30.3 | 86.49% | 5 | *indica* | Bangladesh |
| 84 | Seleccion 138 | A10 | AP8 | 106 | 19.97 | 28.0 | 91.52% | 7 | *indica* | Cuba |
| 85 | Tieu phat | A10 | AP8 | 222 | 26.97 | 16.6 | 84.11% | 9 | *japonica* | Virtnam |
| 86 | IR 4707-106-3-2 | A10 | AP8 | 103 | 16.96 | 20.5 | 83.32% | 10 | *indica* | Philippines |
| 87 | B 995-bci-tb-13 | A9 | AP8 | 107 | 15.90 | 23.5 | 83.42% | 9 | *indica* | Indonesia |
| 89 | Itarare | A5 | AP3 | 120 | 14.65 | 32.5 | 93.63% | 4 | *japonica* | Brazil |
| 90 | IR 9669-pp 846-1 | A12 | AP1 | 96 | 23.11 | 26.0 | 83.81% | 11 | *indica* | Philippines |
| 91 | IR 2071-588-5-1 | A10 | AP8 | 85 | 24.02 | 22.7 | 92.97% | 13 | *indica* | Philippines |
| 92 | IR 2070-834-1-2-2 | A16 | AP10 | 92 | 19.70 | 20.6 | 76.71% | 16 | *indica* | Philippines |
| 93 | Nira prieto | A18 | AP14 | 140 | 17.86 | 25.9 | 92.41% | 5 | *indica* | Cuba |
| 95 | Arroz bolito | A5 | AP3 | 115 | 19.98 | 30.0 | 91.82% | 6 | *japonica* | Cuba |
| 96 | Long ma | A10 | AP8 | 133 | 12.36 | 29.9 | 85.32% | 5 | *indica* | Laos |
| 97 | IR 442-2-50-2-2-3 | A20 | AP13 | 125 | 30.65 | 26.3 | 82.42% | 12 | *indica* | Philippines |
| 98 | Bhog | A5 | AP3 | 114 | 14.42 | 13.5 | 82.56% | 11 | *indica* | India |
| 99 | Quilligama | A9 | AP8 | 151 | 38.10 | 22.0 | 90.08% | 13 | *indica* | Liberia |
| 11001 | Wenxiangru | A2 | AP9 | 111 | 12.2 | 45.4 | 86.67% | 3 | *japonica* | Yunnan |
| 11002 | Babaili | A3 | AP2 | 180 | 4.9 | 26.8 | 85.91% | 6 | *japonica* | Guizhou |
| 11003 | Aizizhan | A8 | AP9 | 79 | 26.3 | 26.6 | 89.61% | 15 | *indica* | Guangdong |
| 11004 | Jiduilun | A14 | AP11 | 110 | 13.4 | 26.7 | 94.17% | 11 | *indica* | Thailand |
| 11006 | Balilla | A1 | AP11 | 107 | 15.8 | 27.0 | 83.55% | 8 | *japonica* | Italy |
| 11007 | Huasizhan | A8 | AP9 | 133 | 23.4 | 24.1 | 86.50% | 10 | *indica* | Guangdong |
| 11008 | Pc311 | A8 | AP9 | 191 | 22.0 | 24.1 | 82.97% | 13 | *japonica* | China |
| 11009 | C418 | A8 | AP9 | 220 | 18.5 | 28.4 | 89.21% | 5 | *japonica* | Liaoning |
| 11010 | Azucena | A7 | AP2 | 122 | 11.4 | 28.3 | 77.13% | 5 | *indica* | China |
| 11011 | Chaoyangzao | A8 | AP9 | 135 | 29.5 | 30.4 | 87.21% | 9 | *indica* | Guangdong |
| 11012 | pzcos | A8 | AP9 | 193 | 24.9 | 23.7 | 74.01% | 8 | *indica* | China |
| 11013 | Fribiprofit | A8 | AP9 | 178 | 17.5 | 22.8 | 73.51% | 6 | *indica* | China |
| 11014 | 9628 | A3 | AP2 | 165 | 15.5 | 29.7 | 85.72% | 5 | *indica* | China |
| 11015 | Aihuangzhong14 | A1 | AP11 | 71 | 24.2 | 29.3 | 95.04% | 13 | *japonica* | Zhejiang |
| 11016 | Nantehao | A8 | AP9 | 88 | 15.8 | 29.5 | 93.86% | 7 | *indica* | Jiangxi |
| 11017 | IR9 | A8 | AP9 | 134 | 26.1 | 32.3 | 94.67% | 7 | *indica* | Guangdong |
| 11019 | Guichao 2 | A8 | AP9 | 167 | 18.1 | 28.7 | 90.93% | 5 | *indica* | Guangxi |
| 11021 | Zaiyeqing | A8 | AP9 | 131 | 18.4 | 22.9 | 94.40% | 7 | *indica* | Guangdong |
| 11022 | Zhihui | A8 | AP9 | 144 | 28.6 | 30.0 | 88.28% | 9 | *indica* | China |
| 11023 | Xiangzaoxian21 | A14 | AP11 | 129 | 27.2 | 33.8 | 87.76% | 7 | *indica* | Hu'nan |
| 11024 | 21 | A8 | AP9 | 129 | 13.7 | 27.0 | 88.04% | 8 | *indica* | China |
| 11026 | Ps09 | A11 | AP11 | 99 | 16.6 | 25.1 | 84.93% | 8 | *indica* | China |
| 11030 | Guanglu'ai | A7 | AP2 | 150 | 19.2 | 27.3 | 93.59% | 5 | *indica* | Guangdong |
| 11031 | Peiai64 | A8 | AP9 | 141 | 15.2 | 22.5 | 85.39% | 8 | *indica* | Hu'nan |
| 11033 | Fenghuazhan | A8 | AP9 | 200 | 23.0 | 21.1 | 78.53% | 7 | *indica* | Guangdong |
| 11035 | Nanzan11 | A8 | AP9 | 150 | 20.0 | 26.5 | 73.43% | 6 | *indica* | Guizhou |
| 11037 | Yangdao6 | A8 | AP9 | 194 | 20.8 | 32.9 | 82.04% | 4 | *indica* | Jiangsu |
| 11038 | Shuhui527 | A8 | AP9 | 206 | 23.1 | 34.4 | 81.43% | 6 | *indica* | Sichuan |
| 11040 | Fengqing'ai | A8 | AP9 | 170 | 25.4 | 25.8 | 84.44% | 8 | *indica* | Guangdong |
| 11041 | Teiqing | A8 | AP9 | 190 | 18.3 | 23.0 | 70.70% | 7 | *indica* | Guangdong |
| 11042 | Yangdao2 | A8 | AP9 | 172 | 20.4 | 28.2 | 68.63% | 7 | *indica* | Jiangsu |
| 11043 | Feng'aizhan1 | A14 | AP11 | 240 | 16.2 | 20.7 | 71.10% | 7 | *indica* | Guangdong |
| 11044 | Qing'guiai5 | A8 | AP9 | 199 | 18.2 | 24.1 | 90.83% | 5 | *indica* | Guangdong |
| 11045 | Jiang'erai | A8 | AP9 | 101 | 19.0 | 27.6 | 91.06% | 8 | *indica* | Guangdong |
| 11046 | Fengbazhan | A8 | AP9 | 126 | 15.7 | 30.2 | 80.43% | 6 | *indica* | Guangdong |
| 11048 | Guangchang'ai | A8 | AP9 | 136 | 17.4 | 22.4 | 80.22% | 10 | *indica* | Guangdong |
| 11049 | Qingsiai | A7 | AP2 | 121 | 11.5 | 21.9 | 69.03% | 17 | *indica* | China |
| 11050 | Guiyang'ai | A8 | AP9 | 119 | 12.3 | 21.6 | 70.86% | 6 | *indica* | Guangdong |
| 11051 | Nanjing6 | A8 | AP9 | 116 | 14.1 | 26.8 | 84.14% | 5 | *indica* | Jiangsu |
| 11052 | Nantehao | A8 | AP9 | 123 | 37.5 | 25.7 | 86.41% | 17 | *indica* | Jiangxi |
| 11053 | Huiyangzhenzhuzao | A7 | AP2 | 169 | 12.1 | 23.5 | 71.65% | 11 | *indica* | Guangdong |
| 11055 | Sizhan | A14 | AP11 | 173 | 13.5 | 19.8 | 53.49% | 13 | *indica* | Guangdong |
| 11056 | Xinan175 | A3 | AP2 | 98 | 17.5 | 30.7 | 58.58% | 13 | *japonica* | Thailand |
| 11057 | Qingliuai | A8 | AP9 | 219 | 22.1 | 23.2 | 82.60% | 8 | *indica* | Guangdong |
| 11058 | Nongken58 | A2 | AP9 | 118 | 23.3 | 26 | 89.00% | 10 | *japonica* | Japan |
| 11059 | Kyoto xu | A3 | AP2 | 30 | 6.3 | 22.6 | 81.73% | 12 | *japonica* | Japan |
| 11060 | *Oryza latifolia Desv* | A7 | AP2 | 79 | 10.5 | 25.8 | 79.56% | 8 | */* | South America |
| 11061 | Taichung Native 1 | A7 | AP2 | 135 | 16.4 | 24.2 | 72.93% | 10 | *indica* | Thailand |
| 11062 | Guhuahuang | A3 | AP2 | 67 | 7.1 | 26.3 | 83.72% | 6 | *japonica* | Jiangsu |
| 11063 | Qingnong'ai | A7 | AP2 | 86 | 3.4 | 25.4 | 88.69% | 9 | *indica* | China |
| 11064 | Balilla | A1 | AP11 | 106 | 14.2 | 25.4 | 81.08% | 7 | *japonica* | Italy |
| 11065 | YTB | A8 | AP9 | 146 | 17.9 | 23.1 | 83.36% | 8 | *indica* | Guangdong |
| 11066 | IR24 | A14 | AP11 | 149 | 18.5 | 29.5 | 79.36% | 6 | *indica* | Philippines |
| 11067 | ZS97B | A8 | AP9 | 85 | 11.7 | 26.0 | 90.52% | 7 | *indica* | Zhejiang |
| 11069 | Haodali | A8 | AP9 | 118 | 17.0 | 38.6 | 76.02% | 6 | *indica* | China |
| 11074 | Nep doi | A4 | AP4 | 67 | / | / | 50.29% | 13 | *indica* | Vietnam |
| 11075 | Nep nuong ro do | A4 | AP4 | 59 | 4.5 | 26.9 | 58.01% | 9 | *indica* | Vietnam |
| 11077 | Nep dai loan | A8 | AP9 | 199 | 16.7 | 20.2 | 63.39% | 7 | *indica* | Vietnam |
| 11079 | Nep badong | A8 | AP9 | 100 | 13.3 | 19.4 | 70.49% | 9 | *indica* | Vietnam |
| 11081 | nep vang ong | A4 | AP4 | 116 | 12.5 | 15.8 | 68.39% | 14 | *indica* | Vietnam |
| 11082 | Cingwulung | A8 | AP9 | 191 | 16.3 | 20.8 | 79.83% | 9 | *indica* | Indonesia |
| 11090 | lekat hutam | A3 | AP2 | 225 | 17.9 | 20.2 | 82.49% | 7 | *indica* | Indonesia |
| 11097 | leuang chaiyaphum | A8 | AP9 | 89 | 12.8 | 26.7 | 67.07% | 9 | *indica* | Thailand |
| 11098 | kampai peum meuang | A8 | AP9 | 124 | 19.5 | 32.4 | 61.68% | 10 | *indica* | Thailand |
| 11099 | khao meuang chon | A13 | AP6 | 111 | 15.3 | 22.1 | 67.33% | 10 | *indica* | Thailand |
| 11100 | khao kai | A8 | AP9 | 99 | 23.8 | 34.3 | 82.91% | 13 | *indica* | Thailand |
| 11103 | Jarey | A7 | AP2 | 125 | 10.3 | 23.5 | 86.21% | 10 | *indica* | Bhutan |
| 11104 | booti | A1 | AP11 | 50 | 6.3 | 22.8 | 59.33% | 7 | *indica* | Pakistan |
| 11106 | Jibun | A2 | AP9 | 149 | 15.8 | 27.1 | 74.86% | 6 | *indica* | Malaysia |
| 11109 | Ganja choota | A8 | AP9 | 150 | 11.0 | 22.3 | 89.75% | 10 | *indica* | Pakistan |
| 11110 | alubis | A4 | AP4 | 157 | 10.8 | 28.7 | 54.87% | 7 | *indica* | Malaysia |
| 11111 | sathiya | A6 | AP7 | 80 | 9.7 | 18.9 | 92.59% | 9 | *indica* | India |
| 11112 | Gembira kuning | A4 | AP4 | 186 | 16.1 | 19.9 | 66.35% | 9 | *indica* | Indonesia |
| 11113 | Dudh malai | A15 | AP4 | 100 | 13.5 | 23.8 | 94.47% | 7 | *indica* | India |
| 11115 | Bishan porag | A8 | AP9 | 115 | 10.1 | 15.7 | 82.97% | 6 | *indica* | India |
| 11116 | Caka putih | A8 | AP9 | 161 | 26.1 | 24.9 | 82.42% | 11 | *indica* | Indonesia |
| 11117 | Gembira kuning | A2 | AP9 | 156 | 24.3 | 22.4 | 74.30% | 12 | *indica* | Indonesia |
| 11118 | Siam hitam | A8 | AP9 | 132 | 28.6 | 22.3 | 88.77% | 13 | *indica* | Indonesia |
| 11119 | Ma goda al | A8 | AP9 | 180 | 28.0 | 26.2 | 84.03% | 10 | *indica* | Sri Lanka |
| 11120 | kohu gamboda | A8 | AP9 | 101 | 15.7 | 22.7 | 70.10% | 8 | *indica* | Sri Lanka |
