# Supplementary material for: Artificial Selection of Gn1a Plays an Important role in Improving Rice Yields Across Different Ecological Regions
Source: Rice (N Y). 2015 Dec 16;8:37. doi: 10.1186/s12284-015-0071-4 (PMC4681714; doi:10.1186/s12284-015-0071-4)
Supplement: Additional file 2: Table S2. — Basic information on the wild rice varieties. The number ahead of each material indicated the serial number in the field in 2012, and the alleles were established based on the different amino acid sequences among the wild rice varieties. (DOC 38 KB) [file 12284_2015_71_MOESM2_ESM.doc]

**Additional file 2: Table S2.**

|  | | | | | | | | | |
| --- | --- | --- | --- | --- | --- | --- | --- | --- | --- |
| **NO.** | **Matierial** | **Allele** | **Specie** |  | **NO.** | **Matierial** | **Allele** | **Specie** | |
| 12001 | Tianyang | AP1 | *O. rufipogon* |  | 12014 | Hong 6 | AP8 | *O. rufipogon* | |
| 12002 | Song 6 | AP8 | *O. rufipogon* |  | 12015 | Bai 1 | AP1 | *O. rufipogon* | |
| 12003 | Yangdong | AP15 | *O. rufipogon* |  | 12016 | Dongxiang 2 | AP1 | *O. rufipogon* | |
| 12005 | Baise | AP16 | *O. rufipogon* |  | 12019 | 16 | AP20 | *O. rufipogon* | |
| 12006 | shuibian | AP17 | *O. rufipogon* |  | 12020 | 18 | AP21 | *O. rufipogon* | |
| 12007 | Tanxie | AP1 | *O. rufipogon* |  | 12021 | 38 | AP22 | *O. rufipogon* | |
| 12008 | W34 | AP18 | *O. rufipogon* |  | 12024 | 46 | AP8 | *O. rufipogon* | |
| 12009 | Wanlingdongxi | AP1 | *O. rufipogon* |  | 12025 | 47 | AP23 | *O. rufipogon* | |
| 12011 | Wenchang | AP1 | *O. rufipogon* |  | 12026 | 53 | AP1 | *O. rufipogon* | |
| 12012 | Meilan | AP19 | *O. rufipogon* |  | 12027 | 56 | AP8 | *O. rufipogon* | |
| 12013 | Dongxi | AP3 | *O. rufipogon* |  |  |  |  | |  |
