# Supplementary material for: Artificial Selection of Gn1a Plays an Important role in Improving Rice Yields Across Different Ecological Regions
Source: Rice (N Y). 2015 Dec 16;8:37. doi: 10.1186/s12284-015-0071-4 (PMC4681714; doi:10.1186/s12284-015-0071-4)
Supplement: Additional file 3: Table S3. — Amino acid variations of the different alleles in O. rufipogon. The number in the first line indicates the variable amino acid sites of the GN1A protein and the GN1A alleles are in brackets; “-” indicates the deletion of the corresponding amino acid, and “&” indicates the stop codon of the protein. (DOC 68 kb) [file 12284_2015_71_MOESM3_ESM.doc]

**Additional file 3: Table S3.**

| Alleles | 38 | 46 | 54 | 79 | 107 | 118 | 150 | 184 | 221 | 230 | 242 | 283 | 295 | 328 | 442 | 462 | 535 | 537 |
| --- | --- | --- | --- | --- | --- | --- | --- | --- | --- | --- | --- | --- | --- | --- | --- | --- | --- | --- |
| 12001 (AP1) | D | --- | A | AAAA | A | R | E | H | EMV | A | G | A | ----- | S | G | NK | G | K |
| 12002 (AP8) | D | --- | A | ---- | A | R | E | H | EMV | A | G | A | ----- | S | G | NK | G | K |
| 12003 (AP15) | D | --- | A | AAAA | A | R | E | H | EMV | A | G | A | ----- | S | G | NK | S | K |
| 12005 (AP16) | D | --- | A | AAAA | A | R | E | H | EMV | S | G | T | GLMDY | S | G | NK | G | K |
| 12006 (AP17) | D | --- | A | AAAA | S | R | E | H | EMV | S | G | T | GLMDY | S | G | NK | G | K |
| 12007 (AP1) | D | --- | A | AAAA | A | R | E | H | EMV | A | G | A | ----- | S | G | NK | G | K |
| 12008 (AP18) | D | DLG | A | AA-- | A | R | E | H | EMV | A | G | D | ----- | S | G | NK | G | K |
| 12009 (AP1) | D | --- | A | AAAA | A | R | E | H | EMV | A | G | A | ----- | S | G | NK | G | K |
| 12011 (AP1) | D | --- | A | AAAA | A | R | E | H | EMV | A | G | A | ----- | S | G | NK | G | K |
| 12012 (AP19) | D | --- | A | A--- | A | R | E | H | EMV | A | R | A | ----- | S | G | NK | G | K |
| 12013 (AP3) | D | --- | A | AAAA | S | R | E | H | EMV | A | G | A | ----- | S | G | NK | G | K |
| 12014 (AP8) | D | --- | A | ---- | A | R | E | H | EMV | A | G | A | ----- | S | G | NK | G | K |
| 12015 (AP1) | D | --- | A | AAAA | A | R | E | H | EMV | A | G | A | ----- | S | G | NK | G | K |
| 12016 (AP1) | D | --- | A | AAAA | A | R | E | H | EMV | A | G | A | ----- | S | G | NK | G | K |
| 12019 (AP20) | D | --- | A | A--- | A | R | E | R | EMV | A | G | A | ----- | S | G | NK | G | K |
| 12020 (AP21) | D | --- | A | AAAA | A | R | E | H | EMV | A | G | A | ----- | S | D | NK | G | K |
| 12021 (AP22) | D | --- | A | ---- | A | R | E | H | EMV | A | G | A | ----- | S | P | Q& |  |  |
| 12024 (AP8) | D | --- | A | ---- | A | R | E | H | EMV | A | G | A | ----- | S | G | NK | G | K |
| 12025 (AP23) | N | --- | A | ---- | A | R | K | H | EMV | A | G | A | ----- | S | G | NK | G | K |
| 12026 (AP1) | D | --- | A | AAAA | A | R | E | H | EMV | A | G | A | ----- | S | G | NK | G | K |
| 12027 (AP8) | D | --- | A | ---- | A | R | E | H | EMV | A | G | A | ----- | S | G | NK | G | K |
