# Supplementary figures and images for: Artificial Selection of Gn1a Plays an Important role in Improving Rice Yields Across Different Ecological Regions
Source: Rice (N Y). 2015 Dec 16;8:37. doi: 10.1186/s12284-015-0071-4 (PMC4681714; doi:10.1186/s12284-015-0071-4)

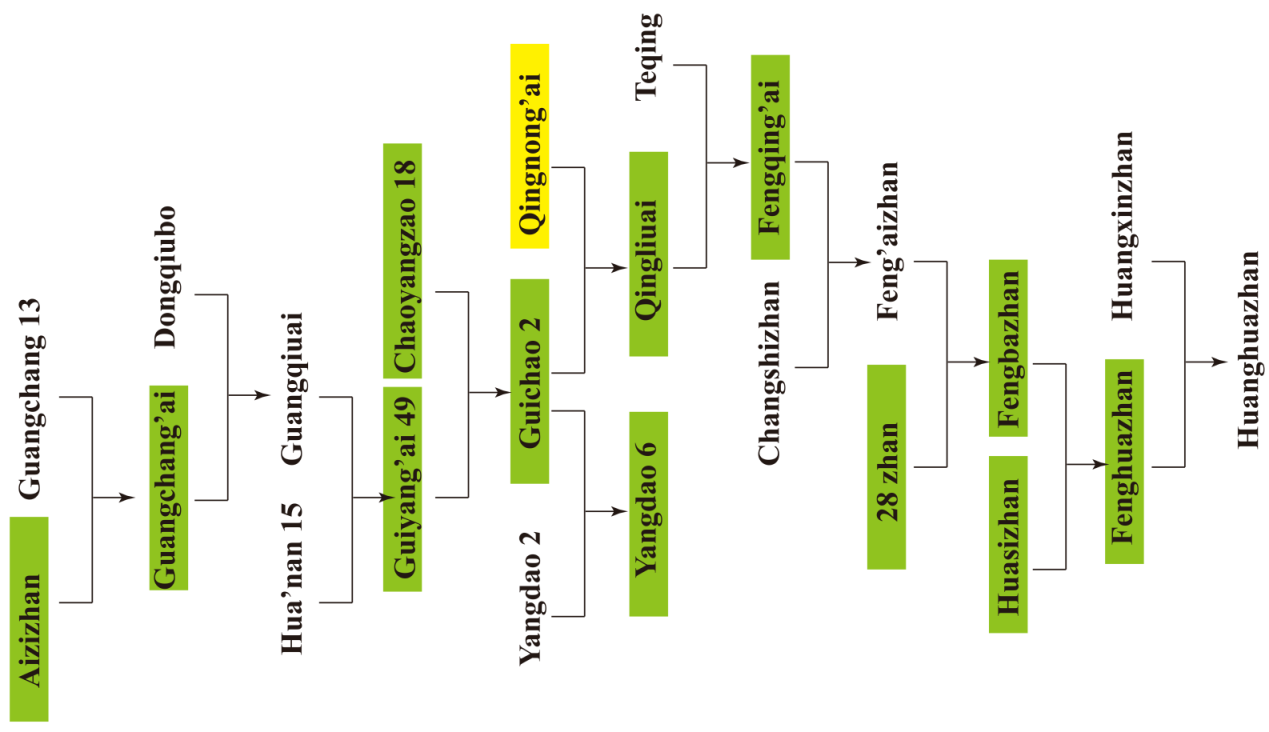


**Additional file 4: Fig. S1.**

Supplement: Additional file 4: Figure S1. — Allele analysis of the pedigree of Guichao 2. The colored materials were selected for this study; the green varieties harbored the AP9 allele and the yellow varieties contained the AP2 allele. (DOC 157 kb) [file 12284_2015_71_MOESM4_ESM.doc]
