# Supplementary material for: Artificial Selection of Gn1a Plays an Important role in Improving Rice Yields Across Different Ecological Regions
Source: Rice (N Y). 2015 Dec 16;8:37. doi: 10.1186/s12284-015-0071-4 (PMC4681714; doi:10.1186/s12284-015-0071-4)
Supplement: Additional file 5: Table S4. — Primers used in this study. (DOC 30 kb) [file 12284_2015_71_MOESM5_ESM.doc]

**Additional file 5: Table S4.**

| **Primers** | **Forward primer** | **Reverse primer** |
| --- | --- | --- |
| Gn1a exon1 | CCTTCCATCGTCAGCACA | AGCACGTTGGAAATCTGG |
| Gn1a exon2 | GCCATGATCGAGGTAATT | TTGCAGCTTAGTTGTTCC |
| Gn1a exon3 | CTTTGCTTGATTTTATTTGC | TGAGGGGTCGTCATTTTG |
| Gn1a exon4 | TGGTGGTAACTAAACATA | ATGCACAGTACACCCATA |
| Gn1a-Promoter | GAGCACTGCCATCCTGAC | TTGAACTCACAACGCACA |
| Actin-RT | TCCATCTTGGCATCTCTCAG | GTACCCTCATCAGGCATCTG |
| Gn1a-RT | GTCCACGACGGCGAGCTCAA | TCATGC GAGT GGTGACGTGA |
